# Introduction of the dengue vaccine in Brazil: the importance of a strengthened Unified Health System in confronting arboviruses

**DOI:** 10.1590/1518-8345.0000.5048

**Published:** 2026-07-31

**Authors:** Ethel Leonor Noia Maciel, Carlos Augusto Grabois Gadelha, Eder Gatti Fernandes, Jadher Percio, Ana Catarina de Melo Araújo, Thayssa Neiva da Fonseca Victer, Rivaldo Venâncio da Cunha, Guilherme Loureiro Werneck, Nísia Trindade Lima

**Affiliations:** 1 Universidade Federal do Espírito Santo, Espírito Santo, ES, Brazil.; 2 Fundação Oswaldo Cruz, Rio de Janeiro, RJ, Brazil.; 3 Ministério da Saúde, Secretaria de Vigilância em Saúde e Ambiente, Departamento do Programa Nacional de Imunizações, Brasília, DF, Brazil.; 4 Fundação Oswaldo Cruz, Campo Grande, MS, Brazil.

**Figure:**
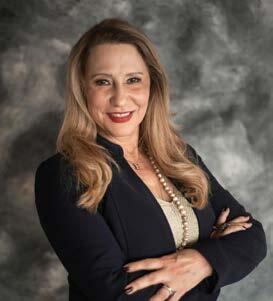

**Figure:**
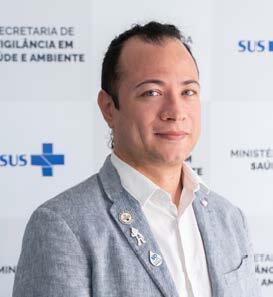

**Figure:**
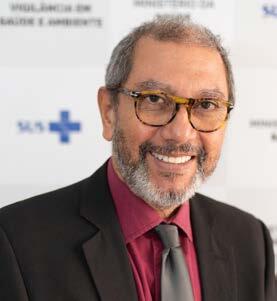

**Figure:**
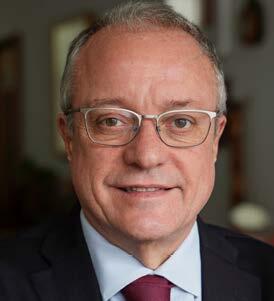

**Figure:**
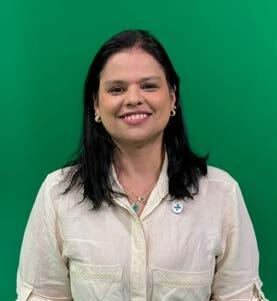

**Figure:**
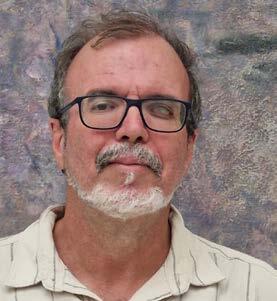

**Figure:**
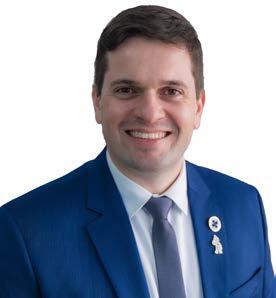

**Figure:**
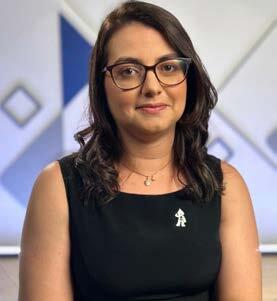

**Figure:**
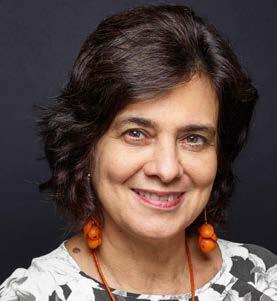

The global incidence of dengue has increased substantially in recent decades, becoming a major public health problem, particularly in countries of the Global South. In Brazil, the widespread dissemination of *Aedes aegypti* and the environmental conditions favorable to its proliferation contribute to the persistence of high transmission rates. The emergence and expansion of the disease are associated with multiple socio-environmental factors, including natural climatic phenomena (such as the 2023 El Niño), climate change with rising temperatures and rainfall, as well as inequalities in access to basic sanitation^1^.

The complexity of this scenario imposes significant challenges to reducing the burden of the disease. In the absence of specific pharmacological measures for prophylaxis and etiological treatment, vector control has historically been consolidated as the main axis of response strategies. However, the mosquito’s high adaptation to urban environments and the persistence of structural determinants limit the effectiveness of these actions, highlighting the need to incorporate other technologies, including vaccines, as essential components of an integrated response^1^.

An ideal dengue vaccine should provide effective and balanced protection against the four viral serotypes (DENV-1 to DENV-4). Currently, three live attenuated tetravalent vaccines are authorized for use in Brazil: CYD-TDV (*Sanofi Pasteur*; multidose schedule), TAK-003/Qdenga (Takeda; two doses), and Butantan-DV (*Instituto Butantan*; single dose, approved in the country at the end of 2025)^2^ ^-^ ^3^. The incorporation of these technologies into the Unified Health System (SUS) represents a milestone in the national immunization policy and positions Brazil as the first country to offer dengue vaccination as a public health action within a universal healthcare system.

Following authorization by the Brazilian Health Regulatory Agency (ANVISA) in March 2023 and the recommendation of the National Commission for the Incorporation of Technologies into the Unified Health System (CONITEC), the TAK-003 vaccine was incorporated into SUS after a rigorous assessment of quality, effectiveness, safety, and cost-benefit for the Brazilian population. This process was conducted within the scope of the National Immunization Program (PNI), created in 1973 and internationally recognized for its technical and organizational capacity, with support from the Technical Advisory Chamber on Immunization (CTAI), responsible for supporting the definition of vaccination strategies.

Given the initial forecast of delivery of 5.1 million doses in 2024, intended for a two-dose primary schedule, children aged 10 to 14 years were established as the initial target population, as this age group presented the highest proportion of dengue hospitalizations, residing in 521 municipalities from health regions classified as priority areas according to epidemiological criteria^4^. After agreement on the proposal within the Tripartite Intermanagerial Commission (CIT), vaccination began on February 9, 2024.

The donation of an additional 1.5 million doses by the manufacturing laboratory made it possible to expand and accelerate the availability of the immunobiological product. By July 3, 2024, 4.1 million doses had been distributed to the states, of which approximately 1.7 million (41%) had been administered. Low adherence and the approaching expiration dates of the doses led the PNI to adopt strategies to avoid waste^5^, including the temporary expansion of vaccination to other age groups, from six to 59 years old. This measure enabled the expansion of the strategy to more than 2,700 municipalities across all regions of the country by the end of 2025.

In 2026, with the approval and introduction into SUS of the nationally produced single-dose Butantan-DV vaccine, vaccination was expanded to individuals aged 15 to 59 years in all Brazilian municipalities, beginning with primary healthcare professionals and later extending to the general population, prioritizing older age groups according to dose availability. Simultaneously, the offer of Qdenga was extended to children and adolescents throughout the national territory. By February 27, 2026, more than 13.5 million doses had been distributed in the country, with more than 3.2 million distributed in the previous six months, and since 2024, more than 6.4 million people had received at least one dose of the dengue vaccine.

The introduction of the Qdenga vaccine in Brazil occurred in a context marked by limitations in the global availability of doses, impacts on costs, international recommendations directed toward endemic areas, and intense social and political pressure amid a large-scale epidemic. Additionally, vaccine hesitancy and the spread of misinformation represent relevant challenges to achieving adequate vaccination coverage^6^. The launch of the National Vaccination Movement in 2023 represented a governmental strategy to restore public confidence and increase vaccine uptake.

At the same time, strengthening national production capacity, with support from the Ministry of Health to *Instituto Butantan* in the development and incorporation of an innovative vaccine^7^, constitutes a central element for the sustainability of the strategy and for reducing dependence on international suppliers.

However, confidence in vaccines constitutes one of the pillars of the PNI. Since 1992, the program has conducted post-authorization pharmacovigilance through monitoring, investigation, and communication of adverse events following immunization. The identification of a safety signal related to the occurrence of anaphylaxis associated with the live attenuated dengue vaccine - a rare event, although more frequent than expected for vaccines in general - demonstrates the robustness of the surveillance system and the response capacity for risk mitigation and assurance of vaccine safety.

The same robust pharmacovigilance framework will be employed in monitoring the introduction of the new dengue vaccine developed by Butantan, allowing for the identification and investigation of serious or unexpected adverse events that might potentially occur. Effective surveillance is one of the fundamental pillars to strengthen and restore public confidence in vaccination.

In summary, dengue vaccination represents an additional and strategic tool in confronting the disease in Brazil, with benefits that outweigh the risks. By ensuring universal and equitable access to immunobiological products, SUS reaffirms its role in reducing health inequalities through a process built upon agreements among the three levels of government. In a scenario of global restrictions on production and innovation, the Brazilian experience demonstrates that strengthening universal health systems and national technological capacity is fundamental to enabling large-scale access and consolidating vaccination as an essential public good.
