# Supplementary material for: Introduction of the dengue vaccine in Brazil: the importance of a strengthened Unified Health System in confronting arboviruses
Source: Rev Lat Am Enfermagem. 2026 Jul 31;34:e5048. doi: 10.1590/1518-8345.0000.5048 (PMC13427256; doi:10.1590/1518-8345.0000.5048)
Supplement: Translation

# Translation: Introdução da vacina contra a dengue no Brasil: a importância de um Sistema Único de Saúde fortalecido no enfrentamento das arboviroses

**DOI:** 10.1590/1518-8345.0000.5049

**Authors:** Ethel Leonor Noia Maciel, Carlos Augusto Grabois Gadelha, Eder Gatti Fernandes, Jadher Percio, Ana Catarina de Melo Araújo, Thayssa Neiva da Fonseca Victer, Rivaldo Venâncio da Cunha, Guilherme Loureiro Werneck, Nísia Trindade Lima

**Affiliations:** 1 Universidade Federal do Espírito Santo, Espírito Santo, ES, Brasil.; 2 Fundação Oswaldo Cruz, Rio de Janeiro, RJ, Brasil.; 3 Ministério da Saúde, Secretaria de Vigilância em Saúde e Ambiente, Departamento do Programa Nacional de Imunizações, Brasília, DF, Brasil.; 4 Fundação Oswaldo Cruz, Campo Grande, MS, Brasil.

A incidência global da dengue aumentou substancialmente nas últimas décadas, configurando-se como um importante problema de saúde pública, sobretudo nos países do Sul Global. No Brasil, a ampla disseminação do *Aedes aegypti* e as condições ambientais favoráveis à sua proliferação contribuem para a persistência de elevadas taxas de transmissão. A emergência e a expansão da doença estão associadas a múltiplos fatores socioambientais, incluindo fenômenos climáticos naturais (como o El Niño de 2023), as mudanças climáticas, com aumento de temperatura e pluviosidade, além das desigualdades no acesso ao saneamento básico^1^.

A complexidade desse cenário impõe desafios significativos à redução da carga da doença. Na ausência de medidas farmacológicas específicas para profilaxia e tratamento etiológico, o controle do vetor consolidou-se historicamente como o principal eixo das estratégias de enfrentamento. Entretanto, a elevada adaptação do mosquito aos ambientes urbanos e a persistência de determinantes estruturais limitam a efetividade dessas ações, evidenciando a necessidade de incorporar outras tecnologias, entre elas as vacinas, como componentes essenciais de uma resposta integrada^1^.

Uma vacina ideal contra a dengue deve conferir proteção eficaz e equilibrada contra os quatro sorotipos virais (DENV-1 a DENV-4). Atualmente, três vacinas tetravalentes de vírus vivos atenuados estão autorizadas para uso no Brasil: CYD-TDV (Sanofi Pasteur; esquema multidose), TAK-003/Qdenga (Takeda; duas doses) e Butantan-DV (Instituto Butantan; dose única, aprovada no país no final de 2025)^2^ ^-^ ^3^. A incorporação dessas tecnologias ao Sistema Único de Saúde (SUS) representa um marco na política nacional de imunizações e posiciona o Brasil como o primeiro país a ofertar a vacinação contra a dengue como ação de saúde pública em um sistema universal.

Após a autorização da Agência Nacional de Vigilância Sanitária (ANVISA), em março de 2023, e a recomendação da Comissão Nacional de Incorporação de Tecnologias no Sistema Único de Saúde (CONITEC), a vacina TAK-003 foi incorporada ao SUS após criteriosa avaliação de qualidade, efetividade, segurança e custo-benefício para a população brasileira. Esse processo foi conduzido no âmbito do Programa Nacional de Imunizações (PNI), criado em 1973 e reconhecido internacionalmente por sua capacidade técnica e organizacional, com apoio da Câmara Técnica Assessora em Imunizações (CTAI), responsável por subsidiar a definição das estratégias de vacinação.

Diante da previsão inicial de entrega de 5,1 milhões de doses em 2024, destinadas a um esquema primário de duas doses, estabeleceu-se como público-alvo inicial crianças de 10 a 14 anos, faixa etária com maior proporção de hospitalizações por dengue, residentes em 521 municípios de regiões de saúde classificadas como prioritárias segundo critérios epidemiológicos^4^. Após a pactuação da proposta na Comissão Intergestores Tripartite (CIT), a vacinação foi iniciada em 9 de fevereiro de 2024.

A doação de 1,5 milhão de doses adicionais pelo laboratório fabricante permitiu ampliar e acelerar a oferta do imunobiológico. Até 3 de julho de 2024, 4,1 milhões de doses haviam sido distribuídas aos estados, das quais aproximadamente 1,7 milhão (41%) foram aplicadas. A baixa adesão e a proximidade do vencimento das doses levaram o PNI a adotar estratégias para evitar perdas^5^, incluindo a ampliação temporária da vacinação para outras faixas etárias, de seis a 59 anos. Essa medida possibilitou expandir a estratégia para mais de 2.700 municípios em todas as regiões do país até o final de 2025.

Em 2026, com a aprovação e a introdução no SUS da vacina Butantan-DV, de dose única e produção nacional, a vacinação foi ampliada para indivíduos de 15 a 59 anos em todos os municípios brasileiros, iniciando-se pelos profissionais da atenção primária à saúde e, posteriormente, pela população geral, com priorização das faixas etárias mais elevadas conforme a disponibilidade de doses. Paralelamente, a oferta da Qdenga foi estendida a crianças e adolescentes em todo o território nacional. Até 27 de fevereiro de 2026, mais de 13,5 milhões de doses haviam sido distribuídas no país, sendo mais de 3,2 milhões nos seis meses anteriores, e, desde 2024, mais de 6,4 milhões de pessoas haviam recebido ao menos uma dose da vacina contra a dengue.

A introdução da vacina Qdenga no Brasil ocorreu em um contexto marcado por limitações na disponibilidade global de doses, impacto nos custos, recomendações internacionais direcionadas a áreas endêmicas e intensa pressão social e política em meio a uma epidemia de grande magnitude. Adicionalmente, a hesitação vacinal e a disseminação de desinformação configuram desafios relevantes para o alcance de coberturas adequadas^6^. O lançamento do Movimento Nacional pela Vacinação, em 2023, representou uma estratégia governamental para recuperar a confiança da população e ampliar a adesão às vacinas.

Paralelamente, o fortalecimento da capacidade produtiva nacional, com o apoio do Ministério da Saúde ao Instituto Butantan no desenvolvimento e na incorporação de uma vacina inovadora^7^, constitui elemento central para a sustentabilidade da estratégia e para a redução da dependência de fornecedores internacionais.

No entanto, a confiança nas vacinas constitui um dos pilares do PNI. Desde 1992, o programa realiza a farmacovigilância pós-autorização por meio do monitoramento, investigação e comunicação dos eventos adversos pós-vacinação. A identificação de um sinal de segurança relacionado à ocorrência de anafilaxia associada à vacina atenuada contra a dengue, evento raro, porém mais frequente do que o esperado para vacinas em geral, demonstra a robustez do sistema de vigilância e a capacidade de resposta para mitigação de riscos e garantia da segurança vacinal.

A mesma estrutura robusta de farmacovigilância será empregada no monitoramento da introdução da nova vacina contra a dengue desenvolvida pelo Butantan, permitindo a identificação e a investigação de eventos adversos graves ou inesperados que eventualmente possam ocorrer. Uma vigilância eficaz é um dos pilares fundamentais para fortalecer e recuperar a confiança da população na vacinação.

Em síntese, a vacinação contra a dengue representa uma ferramenta adicional e estratégica no enfrentamento da doença no Brasil, com benefícios que superam os riscos. Ao garantir acesso universal e equitativo aos imunobiológicos, o SUS reafirma seu papel na redução das desigualdades em saúde, em um processo construído por meio de pactuações entre as três esferas de governo. Em um cenário de restrições globais de produção e inovação, a experiência brasileira evidencia que o fortalecimento de sistemas universais de saúde e da capacidade tecnológica nacional é fundamental para viabilizar o acesso em larga escala e consolidar a vacinação como um bem público essencial.

# Translation: Introducción de la vacuna contra el dengue en Brasil: la importancia de un Sistema Único de Salud fortalecido en el enfrentamiento de las arbovirosis

**DOI:** 10.1590/1518-8345.0000.5047

**Authors:** Ethel Leonor Noia Maciel, Carlos Augusto Grabois Gadelha, Eder Gatti Fernandes, Jadher Percio, Ana Catarina de Melo Araújo, Thayssa Neiva da Fonseca Victer, Rivaldo Venâncio da Cunha, Guilherme Loureiro Werneck, Nísia Trindade Lima

**Affiliations:** 1 Universidade Federal do Espírito Santo, Espírito Santo, ES, Brasil.; 2 Fundação Oswaldo Cruz, Rio de Janeiro, RJ, Brasil.; 3 Ministério da Saúde, Secretaria de Vigilância em Saúde e Ambiente, Departamento do Programa Nacional de Imunizações, Brasília, DF, Brasil.; 4 Fundação Oswaldo Cruz, Campo Grande, MS, Brasil.

La incidencia global del dengue ha aumentado sustancialmente en las últimas décadas, configurándose como un importante problema de salud pública, especialmente en los países del Sur Global. En Brasil, la amplia diseminación del *Aedes aegypti* y las condiciones ambientales favorables para su proliferación contribuyen a la persistencia de elevadas tasas de transmisión. La emergencia y expansión de la enfermedad están asociadas a múltiples factores socioambientales, incluyendo fenómenos climáticos naturales (como El Niño de 2023), el cambio climático con aumento de temperatura y precipitaciones, además de las desigualdades en el acceso al saneamiento básico^1^.

La complejidad de este escenario impone desafíos significativos para la reducción de la carga de la enfermedad. En ausencia de medidas farmacológicas específicas para la profilaxis y el tratamiento etiológico, el control vectorial se ha consolidado históricamente como el principal eje de las estrategias de enfrentamiento. Sin embargo, la elevada adaptación del mosquito a los ambientes urbanos y la persistencia de determinantes estructurales limitan la efectividad de estas acciones, evidenciando la necesidad de incorporar otras tecnologías, entre ellas las vacunas, como componentes esenciales de una respuesta integrada^1^.

Una vacuna ideal contra el dengue debe conferir protección eficaz y equilibrada contra los cuatro serotipos virales (DENV-1 a DENV-4). Actualmente, tres vacunas tetravalentes de virus vivos atenuados están autorizadas para uso en Brasil: CYD-TDV (*Sanofi Pasteur*; esquema multidosis), TAK-003/Qdenga (Takeda; dos dosis) y Butantan-DV (*Instituto Butantan*; dosis única, aprobada en el país a finales de 2025)^2^ ^-^ ^3^. La incorporación de estas tecnologías al Sistema Único de Salud (SUS) representa un hito en la política nacional de inmunizaciones y posiciona a Brasil como el primer país en ofrecer la vacunación contra el dengue como acción de salud pública en un sistema universal.

Tras la autorización de la Agencia Nacional de Vigilancia Sanitaria (ANVISA) en marzo de 2023 y la recomendación de la Comisión Nacional de Incorporación de Tecnologías al Sistema Único de Salud (CONITEC), la vacuna TAK-003 fue incorporada al SUS después de una rigurosa evaluación de calidad, efectividad, seguridad y costo-beneficio para la población brasileña. Este proceso fue conducido en el ámbito del Programa Nacional de Inmunizaciones (PNI), creado en 1973 y reconocido internacionalmente por su capacidad técnica y organizacional, con apoyo de la Cámara Técnica Asesora en Inmunizaciones (CTAI), responsable de subsidiar la definición de las estrategias de vacunación.

Ante la previsión inicial de entrega de 5,1 millones de dosis en 2024, destinadas a un esquema primario de dos dosis, se estableció como población objetivo inicial a niños de 10 a 14 años, grupo etario con la mayor proporción de hospitalizaciones por dengue, residentes en 521 municipios de regiones de salud clasificadas como prioritarias según criterios epidemiológicos^4^. Después de la aprobación de la propuesta en la Comisión Intergestora Tripartita (CIT), la vacunación comenzó el 9 de febrero de 2024.

La donación de 1,5 millones de dosis adicionales por parte del laboratorio fabricante permitió ampliar y acelerar la oferta del inmunobiológico. Hasta el 3 de julio de 2024, se habían distribuido 4,1 millones de dosis a los estados, de las cuales aproximadamente 1,7 millones (41%) habían sido aplicadas. La baja adhesión y la proximidad del vencimiento de las dosis llevaron al PNI a adoptar estrategias para evitar pérdidas^5^, incluyendo la ampliación temporal de la vacunación a otros grupos etarios, de seis a 59 años. Esta medida permitió expandir la estrategia a más de 2.700 municipios en todas las regiones del país hasta finales de 2025.

En 2026, con la aprobación e introducción en el SUS de la vacuna Butantan-DV, de dosis única y producción nacional, la vacunación fue ampliada a individuos de 15 a 59 años en todos los municipios brasileños, comenzando por los profesionales de atención primaria de salud y posteriormente por la población general, priorizando los grupos etarios de mayor edad según la disponibilidad de dosis. Paralelamente, la oferta de Qdenga fue extendida a niños y adolescentes en todo el territorio nacional. Hasta el 27 de febrero de 2026, más de 13,5 millones de dosis habían sido distribuidas en el país, siendo más de 3,2 millones en los seis meses anteriores y, desde 2024, más de 6,4 millones de personas habían recibido al menos una dosis de la vacuna contra el dengue.

La introducción de la vacuna Qdenga en Brasil ocurrió en un contexto marcado por limitaciones en la disponibilidad global de dosis, impacto en los costos, recomendaciones internacionales dirigidas a áreas endémicas e intensa presión social y política en medio de una epidemia de gran magnitud. Adicionalmente, la hesitación vacunal y la difusión de desinformación configuran desafíos relevantes para alcanzar coberturas adecuadas^6^. El lanzamiento del Movimiento Nacional por la Vacunación, en 2023, representó una estrategia gubernamental para recuperar la confianza de la población y ampliar la adhesión a las vacunas.

Paralelamente, el fortalecimiento de la capacidad productiva nacional, con el apoyo del Ministerio de Salud al Instituto Butantan en el desarrollo e incorporación de una vacuna innovadora^7^, constituye un elemento central para la sostenibilidad de la estrategia y para la reducción de la dependencia de proveedores internacionales.

Sin embargo, la confianza en las vacunas constituye uno de los pilares del PNI. Desde 1992, el programa realiza farmacovigilancia posautorización mediante el monitoreo, investigación y comunicación de eventos adversos posteriores a la vacunación. La identificación de una señal de seguridad relacionada con la ocurrencia de anafilaxia asociada a la vacuna atenuada contra el dengue -evento raro, aunque más frecuente de lo esperado para vacunas en general- demuestra la solidez del sistema de vigilancia y la capacidad de respuesta para la mitigación de riesgos y garantía de la seguridad vacunal.

La misma estructura robusta de farmacovigilancia será empleada en el monitoreo de la introducción de la nueva vacuna contra el dengue desarrollada por el Butantan, permitiendo la identificación y la investigación de eventos adversos graves o inesperados que eventualmente puedan ocurrir. Una vigilancia eficaz es uno de los pilares fundamentales para fortalecer y recuperar la confianza de la población en la vacunación.

En síntesis, la vacunación contra el dengue representa una herramienta adicional y estratégica en el enfrentamiento de la enfermedad en Brasil, con beneficios que superan los riesgos. Al garantizar acceso universal y equitativo a los inmunobiológicos, el SUS reafirma su papel en la reducción de las desigualdades en salud, en un proceso construido mediante acuerdos entre las tres esferas de gobierno. En un escenario de restricciones globales de producción e innovación, la experiencia brasileña evidencia que el fortalecimiento de sistemas universales de salud y de la capacidad tecnológica nacional es fundamental para viabilizar el acceso a gran escala y consolidar la vacunación como un bien público esencial.
